# Optimizing an Ex Vitro RUBY-Equipped Method for Hairy Root Transformation of Peanuts: An Efficient Approach for the Functional Study of Genes in Peanut Roots

**DOI:** 10.3390/genes16121401

**Published:** 2025-11-24

**Authors:** Xinyue Li, Jun Zhou, Fei Kong, Xiaoyun Li, Dong Xiao, Aiqin Wang, Longfei He, Jie Zhan

**Affiliations:** 1 College of Agriculture, Guangxi University, Nanning 530004, China; 2 College of Life Science and Technology, Guangxi University, Nanning 530004, China; 3 Guangdong Provincial Key Laboratory of Biotechnology for Plant Development, School of Life Sciences, South China Normal University, Guangzhou 510631, China

**Keywords:** peanut, hairy root transformation, RUBY-equipped, *AhLRX6*

## Abstract

*Agrobacterium* *rhizogenes* (*A. rhizogenes*)-mediated transformation of hairy roots is a favored and flexible method for root gene functional analysis. However, the selection of transformants can be complex and time-consuming. Here, we describe our simplified method for the *A. rhizogenes*-mediated hairy root induction in young peanut shoots using an expression vector with *RUBY* for direct visual selection of transformants. Analyses verified that this method provides a high-efficiency gene transformation technique for peanut, with transformant frequencies between 46.2 and 73.7%. To test the utility of this method in gene functional analyses, it was used to overexpress *AhLRX6* in hairy roots and we present our preliminary results indicating the production of thicker cells walls in root tips relative to the WT.

## 1. Introduction

Peanut (*Arachis hypogaea* L.) is an essential annual crop widely planted in semi-arid tropical regions of Asia, Africa, and the Americas, which is a rich source of vegetable oil, proteins, minerals and vitamins. Both the yield and quality of the crop are heavily limited by a range of biotic and abiotic stress factors, including pathogens and insect pests, drought, salinity, and aluminum toxicity, all of which can result in serious economic losses [1]. The discovery of genes involved in responding to biotic/abiotic stresses enhances our knowledge of not only the molecular mechanisms involved but also the molecular targets for use in breeding and bioengineering approaches for peanut improvement [2]. However, the low efficiency of peanut transformation methods, the instability of the transgenic lines produced, and the time-consuming procedures involved have markedly limited progress in gene functional studies in this important crop.

In recent decades, hairy root culture, which uses *Agrobacterium rhizogenes* (*A. rhi zogenes*)-mediated transformation, has been applied successfully for the efficient production of useful metabolites in numerous plant species [3,4]. The development of a highly efficient genetic transformation system for peanut via *A. rhizogenes* would not only facilitate the analysis of root gene functions and precision breeding but also the production of metabolites from peanut hairy roots.

Leucine-rich repeat extensions (LRXs) are categorized as extracellular proteins. They have been found in several plant species and are thought to play important signaling roles in the regulation of cell wall formation during development and the response to abiotic stress [5,6]. We evaluated an ex vitro method for hairy root transformation to obtain transgenic peanut hairy roots overexpressing *AhLRX6*. The method utilizes *RUBY* as the reporter gene that converts tyrosine to vivid red betalain [7,8], which can be directly observed with the naked eye. Here, we present our evaluation of this simplified method for the transformation of peanut seedlings and discuss our results with its use for the overexpression of *AhLRX6* during *Agrobacterium*-induced hairy root formation.

## 2. Materials and Methods

### 2.1. Cultivation of Peanuts

Peanut seeds (cultivar: Zhonghua 34, cultivated variety, National Registration in 2022, Registration No. GPD Peanut (2022) 420137) were incubated in moist perlite in the dark at 26 ± 2 °C for roughly 3–4 days. Germinated seedlings were then transferred to Hoagland’s nutrient solution, with cultivation conditions set as follows: a 16 h light/8 h dark cycle and a photon flux density of 30–50 µmol/m^2^/s at 26 ± 2 °C.

### 2.2. Vector Construction and Transformation

**Figure 1:**
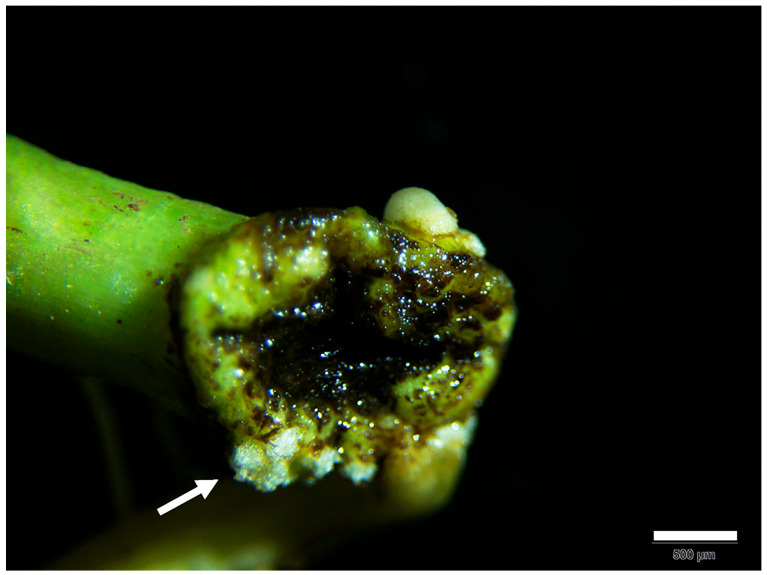
Phenotype of peanut plants. Callus tissue was observed at the incision site after seven days (bar, 500 µm). The white arrow indicates the formation of callus tissue.

The *AhLRX6* overexpression vector was constructed from the 35S:RUBY plasmid, using restriction sites *Pst* I and *Nco* I. The recombinant plasmid was transformed into *A. rhizogene* strain K599 by the heat shock method. Colonies formed on the plate were selected and cultured overnight at 28 °C 250 RPM in TY liquid medium containing 50 mM streptomycin and 50 mM kanamycin. A 100 mL aliquot of this overnight culture was added to 100 mL TY liquid medium containing 10 mM MgCl_2_, 10 mM 2-morpholinoethanesulphonic acid, and 100 μM acetosyringone and allowed to grow until an OD_600_ of 0.8. The plant transformation employed the dipping method of Nanjareddy et al. [9] but was further optimized as follows: the epicotyls peanut seedlings at the two-leaf stage were diagonally excised with a sterile scalpel and placed in the *A. rhizogenes* solution for 30 min. The inoculated seedlings were dried and then transplanted to the matrix soil (matrix soil: vermiculite = 1:1) in the dark for three days before their transfer to a growth chamber at 26 °C with a 16 h light/8 h dark photoperiod. Plants were irrigated every other day with Hoagland nutrient solution and sprayed with sterilized distilled water. After seven days, the formation of callus tissue was observed at the incision site (Figure 1), and the plants were re-inoculated with *A. rhizogenes*.

### 2.3. RT-qPCR Analysis of Peanut Root and Leaf

Total RNA was isolated from peanut hairy roots and leaves using the Eastep total RNA extraction kit (Promega, Shanghai, China) according to the supplier’s recommendations with a 7500 Real Time PCR System and ChamQ Universal SYBR qPCR Master Mix (Novizan Biotechnology Co., Nanjing, China). All primers used for PCR amplification were designed using Primer 5.0 software (Table S1). The 2^−∆∆Ct^ method was used to determine the expression level of each gene relative to *AhUBQR10* (accession no. EG030441), the internal reference [10].

### 2.4. Transmission Electron Microscopy of Root Tips

Root tips (1–2 mm) were excised and fixed in 3% glutaraldehyde over 2 h. The fixed root tips were rinsed three times with 0.1 M PBS, post-fixed in 1% osmium acid, and re-rinsed three times. The segments were subsequently dehydrated using a gradient of ethanol and acetone solutions: 50%, 70%, and 90% ethanol; a 1:1 mixture of 90% ethanol and 90% acetone; 90% acetone; and 100% acetone. The dehydrated segments were permeated successively with acetone: epoxy resin mixtures in a 2:1, 1:2 ratio, followed by 100% epoxy resin. The segments were then placed in epoxy resin (No. 31185, Sigma-Aldrich, St. Louis, MO, USA) for embedding, sectioned at 800–900 Å, treated with double staining (uranyl acetate and lead citrate), mounted on copper grids, and examined by means of transmission electron microscopy (JEOL JEM-1200EX, Akishima, Japan). The average thickness of the cell walls was counted using Image Pro Plus software (Image pro plus 6.0, Media Cybernetics, Rockville, MD, USA), and SPSS Statistics (SPSS 24.0; International Business Machines Corporation, Armonk, NY, USA) was used to analyze data for significance.

## 3. Results

**Figure 2:**
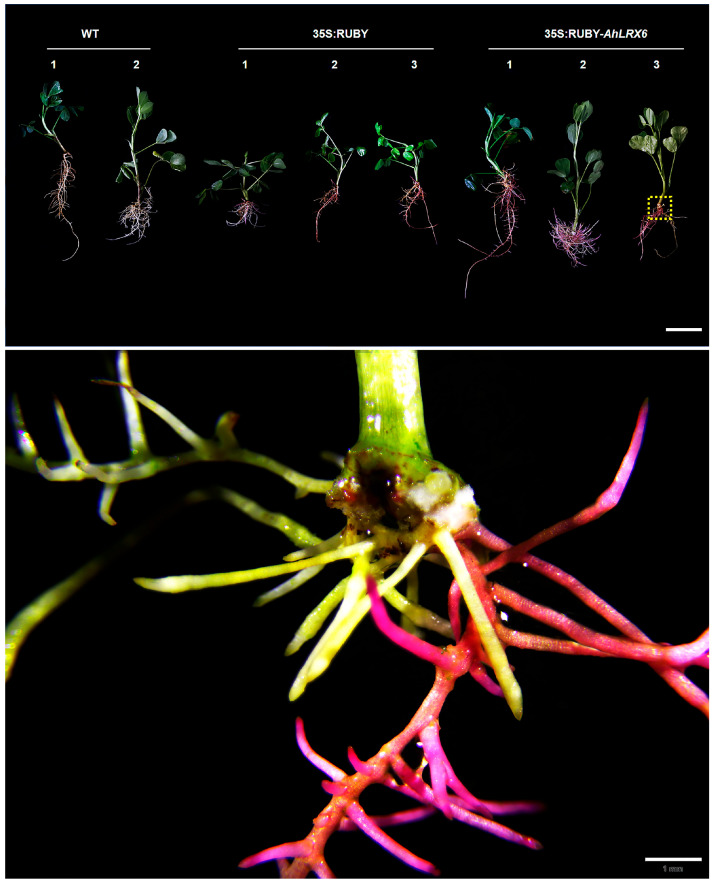
Phenotypes of transgenic-positive hairy roots show *RUBY* gene expression after thirty days (bar, 6 cm). Numbers 1-3 indicate different strains.The figure below is at higher magnification (bar, 1 mm).

Transgenic-positive seedlings could be distinguished ten days post-inoculation by RUBY red coloration near the wound site. Hairy root growth was observed after fourteen days. Mature transgenic hairy roots were observed after thirty days (Figure 2).

**Figure 3:**
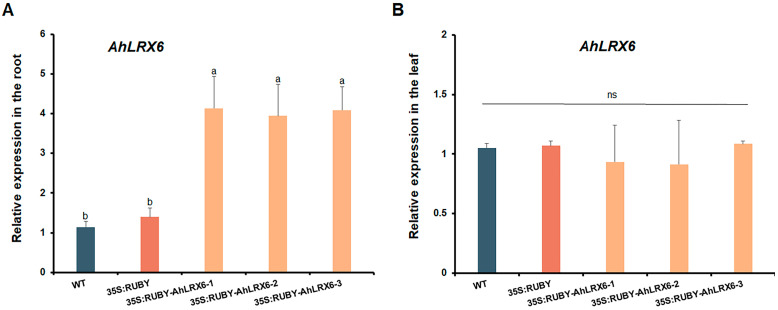
RT-qPCR analysis of root (**A**) and leaf (**B**) *AhLRX6* transcripts. WT and 35S:RUBY represent the negative control seedlings transformed with *A. tumefaciens* strain K599 and the same strain harboring the empty 35S:RUBY vector, respectively. RUBY-AhLRX6 indicates the seedling were transformed for the overexpression of the RUBY: *AhLRX6* fusion product. Lowercase letters represent *p* < 0.05, respectively. Different letters indicate significant differences, evaluated by one-way ANOVA.

In total, hairy roots were successfully induced in 15/29 seedlings (51.7%). On these plants, transgenic-positive hairy roots could be visually selected as early as ten days after transplantation to vermiculite by their red betalain production, indicating a transformation efficiency ranging from 46.2 to 73.7%. The overexpression of *AhLRX6* in transgenic peanut hairy roots was confirmed using RT-qPCR. The results indicate that *AhLRX6* expression in transgenic hairy roots was significantly higher relative to that in either the WT or those transformed with the empty 35S:RUBY vector (Figure 3A), but as expected, the expression of *AhLRX6* remained unaltered in leaves (Figure 3B). We further examined the expression of the *RUBY* gene, and the results are presented in Supplemental Figure S1. No *RUBY* expression was detected in WT plants. In contrast, *RUBY* expression was successfully detected in both the 35S:RUBY empty vector lines (seedlings transformed with the empty 35S:RUBY vector) and the 35S:RUBY-*AhLRX6-1*, *-2*, and *-3* lines (seedlings transformed to overexpress the RUBY:AhLRX6 fusion protein), with no significant differences observed among these lines. These results confirm that the observed changes in *AhLRX6* gene expression are attributed to the transformation process.

**Figure 4:**
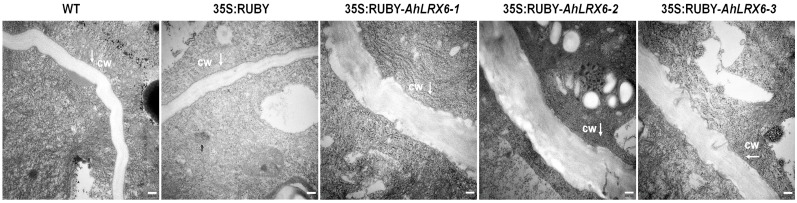
Transmission electron micrographs of peanut hairy root tips. The lines examined are indicated above each panel and are as in Figure 2. Bar, 200 nm. All experiments were repeated with three biological replicates. White arrows indicate the cell wall (CW).

**Figure 5:**
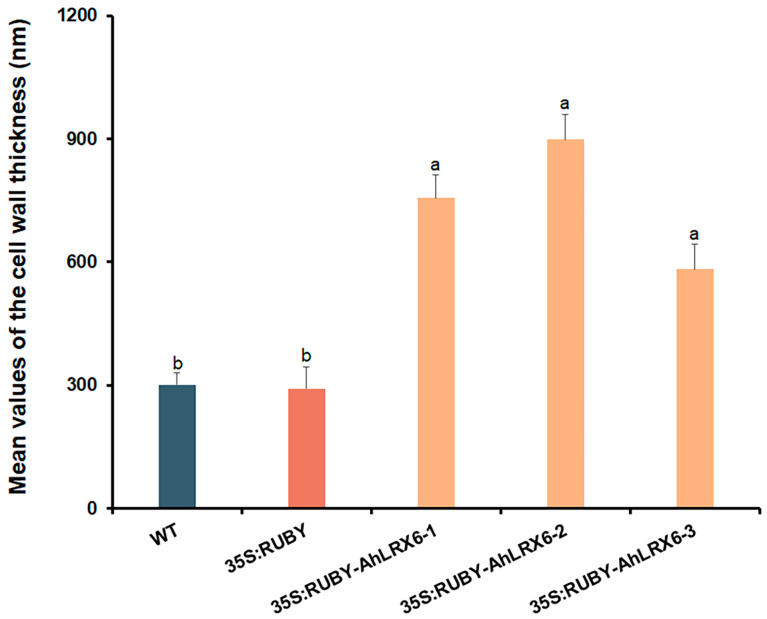
Thickness of root tip cells walls relative to the WT. The lines examined are indicated above each panel and are as in Figure 2. Lowercase letters represent *p* < 0.05, respectively. Different letters indicate significant differences, evaluated by one-way ANOVA. All experiments were repeated with three biological replicates.

In addition, electron microscopy ultrastructural analysis showed that the overexpression of *AhLRX6* in hairy roots resulted in substantially thicker cells walls relative to the WT and those transformed with the empty 35S:RUBY vector (Figure 4 and Figure 5). The data suggest that this system provides a highly effective tool for the analysis of gene functions in peanut roots.

## 4. Discussion

Various reporter genes have been created for plant gene expression research, and β-GUS staining has been broadly utilized in monitoring the patterns of gene expression [11]. Studies have utilized *A. rhizogenes* K599 containing the pCAMBIA1300 vector with the synthetic proDR5::GUS (DR5::GUS promoter) to generate transgenic hairy roots in *A. hypogaea* with a strain utilized in earlier experiments [9,12,13]. It was found that the proDR5::GUS transgenic hairy roots showed robust GUS expression, providing an efficient way to analyze promoter function. However, the processes of dye decolorization and enzyme activity detection in GUS staining can be tedious [14]. GFP has also been utilized as a reporter in the peanut hairy root system [9,13]. While sensitive and accurate, the GFP assay requires special detection equipment, such as a luminometer or imaging system. The present approach involves infecting wound locations with *A. rhizogenes* gel, along with the use of the RUBY vector to visually detect and select positively transformed hairy roots. Compared to these reporter systems, the use of the RUBY reporter allows for the real-time monitoring of betalain content, with no need for costly imaging technologies [15].

Tissue culture-based transformation methods have been developed for hairy root production from leaf [13,16], embryonic axis [17,18], and petiole [12]. However, a highly sterile environment and labor-intensive operations are indispensable for this approach. Nanjareddy et al. [9] demonstrated an approach to hairy root induction from young shoots mediated by *A. rhizogenes*: the cut end of the explant was gently scraped across a freshly prepared cell mat, and the inoculated ends were immersed in B&D media-moistened sterile vermiculite in sealed tissue culture jars and incubated with a 16 h light/8 h dark photoperiod for 10–12 days. To prepare B&D nutrient solution, add 0.5 mL of each stock solution (I, II, III, IV) to 1 L sterile distilled water. Next, incorporate 5 mL of 1 M KNO_3_ and stir constantly with a magnetic mixer to prevent the formation of precipitates. Here, cut epicotyls peanut seedlings were directly dipped into *A. rhizogenes* solution for 30 min. Once the inoculated seedlings were dry, they were transplanted to the matrix soil (matrix soil–vermiculite = 1:1) in the dark for three days before transfer to a 16 h light/8 h dark photoperiod growth chamber. Plants were irrigated with just Hoagland nutrient solution. The formation of callus tissue was observed at the incision site after the same seven days [9]. Compared to these reported peanut hairy root systems, the method described here requires no separate co-culture and only simple experimental procedures.

Previous molecular studies have recognized peanut hairy root cultures as a feasible alternative for producing commercially valuable secondary metabolites [16]. Transformed peanut hairy roots have also been used to investigate genes associated with peanut root nodule symbiosis [17,18], plant–nematode interactions in its detached leaves [13,19], drought resistance [12], and peanut arbuscular mycorrhizal (AM) symbiosis [9]. Furthermore, with global environmental pollution and ecological imbalance growing more prominent, this transformation method allows for the study of gene function under stress conditions, exhibiting great potential in environmental stress response.

In conclusion, we successfully optimized and simplified an ex vitro method for generating peanut hairy root lines with high efficiency. This method has the following advantages: (1) the RUBY reporter allows naked-eye selection and analysis of transformants; (2) high success rate (it demonstrates transformation efficiency of 46.2–73.7%); (3) simple experimental procedure (it only requires cutting–dipping); (4) low cost (it requires only basic plant growth medium components of soil and vermiculite); and (5) applicability to various rapid gene functional analyses, such as for changes in root cell structure. This method offers a robust and flexible tool that we expect will facilitate gene functional analyses in peanut.

## Data Availability

The original contributions presented in the study are included in the article; further inquiries can be directed to the corresponding author.

## Supplementary Materials

The following supporting information can be downloaded at https://www.mdpi.com/article/10.3390/genes16121401/s1: Supplemental Figure S1. RT-qPCR analysis of *Ruby* transcripts. Uppercase and lowercase letters represent *p* < 0.01 and *p* < 0.05, respectively. Different letters indicate significant differences, evaluated by one-way ANOVA. Supplemental Table S1. The primers used in this study.
