# Supplementary material for: Optimizing an Ex Vitro RUBY-Equipped Method for Hairy Root Transformation of Peanuts: An Efficient Approach for the Functional Study of Genes in Peanut Roots
Source: Genes (Basel). 2025 Nov 24;16(12):1401. doi: 10.3390/genes16121401 (PMC12732845; doi:10.3390/genes16121401)
Supplement: Supplementary file 1 [file genes-16-01401-s001.zip › genes-3979002-supplementary Table.pdf]

Table S1 PCR primers in this study

Name	Sequence (5'→3')
RUBY-F	ATGGATCATGCGACCCTC
RUBY-R	TCACTATCACTGGAGGCTTG
35S:RUBY-AhLRX6-F	ctatttacaattacactgcagATGTCAATTCTTACAAAAACCATTCACC
35S:RUBY-AhLRX6-R	cgagggtcgcatgatccatggACTCGAATTATTGTACCCAAAAGCG
q AhLRX6-F	GTATACATGACCGGTCCACCC
q AhLRX6-R	CGCAGAAGATCAAGAAGCCAC
Q AhUBQ10-F	CGCACACTCGCTGACTACAAC
Q AhUBQ10-R	CACGGAGACGGAGGACAAGG
