# Supplementary figures and images for: Optimizing an Ex Vitro RUBY-Equipped Method for Hairy Root Transformation of Peanuts: An Efficient Approach for the Functional Study of Genes in Peanut Roots
Source: Genes (Basel). 2025 Nov 24;16(12):1401. doi: 10.3390/genes16121401 (PMC12732845; doi:10.3390/genes16121401)

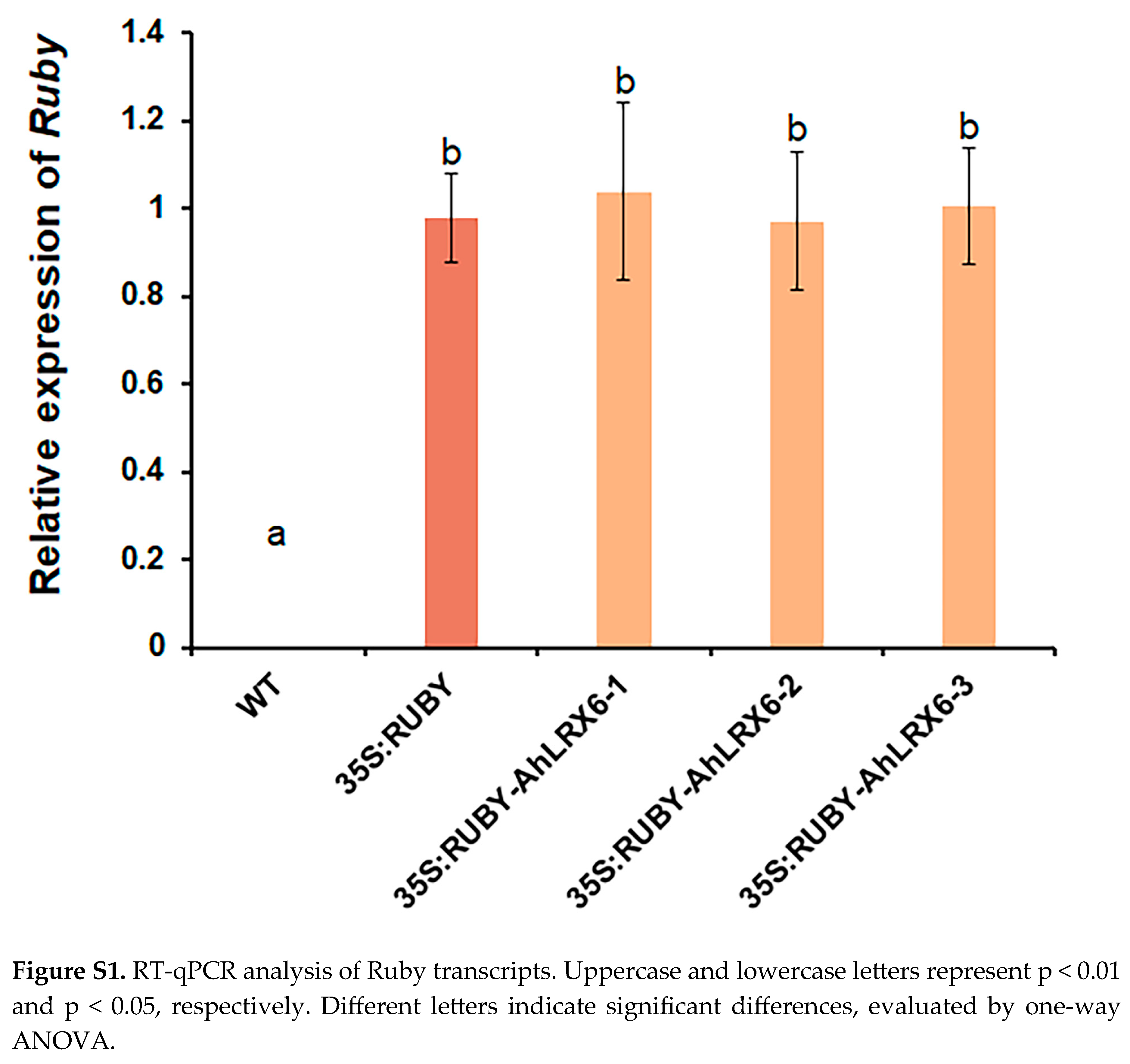

Supplement: Supplementary file 1 [file genes-16-01401-s001.zip › genes-3979002-supplementary Figure.tif]
